# The effect of scoliotic deformity on spine kinematics in adolescents

**DOI:** 10.1186/s13013-016-0103-x

**Published:** 2016-10-25

**Authors:** Sarah Galvis, Douglas Burton, Brandon Barnds, John Anderson, Richard Schwend, Nigel Price, Sara Wilson, Elizabeth Friis

**Affiliations:** 1 Mechanical Engineering and Bioengineering, University of Kansas, 1530 W 15th Street, Lawrence, KS 66045 USA; 2 Department of Orthopedic Surgery, University of Kansas Medical Center, 3901 Rainbow Blvd, Kansas City, KS 66103 USA; 3 Department of Pediatric Orthopedic Surgery, Children’s Mercy Hospital, 2401 Gillham Road, Kansas City, MO 64108 USA

**Keywords:** Adolescent idiopathic scoliosis, Spinal mobility, Thoracic spine, Motion analysis, Kinematics

## Abstract

**Background:**

While adolescent idiopathic scoliosis (AIS) produces well characterized deformation in spinal form, the effect on spinal function, namely mobility, is not well known. Better understanding of scoliotic spinal mobility could yield better treatment targets and diagnoses. The purpose of this study was to characterize the spinal mobility differences due to AIS. It was hypothesized that the AIS group would exhibit reduced mobility compared to the typical adolescent (TA) group.

**Methods:**

Eleven adolescents with right thoracic AIS, apices T6-T10, and eleven age- and gender-matched TAs moved to their maximum bent position in sagittal and coronal plane bending tasks. A Trakstar (Ascension Technologies Burlington, VT) was used to collect position data. The study was approved by the local IRB. Using MATLAB (MathWorks, Natick, MA) normalized segmental angles were calculated for upper thoracic (UT) from T1-T3, mid thoracic (MT) from T3-T6, lower thoracic (LT) from T6-T10, thoracolumbar (TL) from T10-L1, upper lumbar (UL) from L1-L3, and thoracic from T1-L1 by subtracting the standing position from the maximum bent position and dividing by number of motion units in each segment. Mann Whitney tests (α = 0.05) were used to determine mobility differences.

**Results:**

The findings indicated that the AIS group had comparatively increased mobility in the periapical regions of the spine. The AIS group had an increase of 1.2° in the mid thoracic region (*p* = 0.01) during flexion, an increase of 1.0° in the mid thoracic region (*p* = 0.01), 1.5° in the thoracolumbar region (*p* = 0.02), and 0.7° in thoracic region (*p* = 0.04) during left anterior-lateral flexion, an increase of 6.0° in the upper lumbar region (*p* = 0.02) during right anterior-lateral flexion, and an increase of 2.2° in the upper lumbar region during left lateral bending (*p* < 0.01).

**Conclusions:**

Participants with AIS did not have reduced mobility in sagittal or coronal motion. Contrarily, the AIS group often had a greater mobility, especially in segments directly above and below the apex. This indicates the scoliotic spine is flexible and may compensate near the apex.

## Background

While adolescent idiopathic scoliosis (AIS) produces well characterized deformation in spinal form, the effect on spinal function, specifically mobility, is not well characterized. A better understanding on the effect of AIS on spinal mobility could yield better treatment targets and earlier diagnostic means. Therefore, it is important to identify and quantify the mobility disparities caused by AIS. While this area of research is important, very little has focused on the spinal mobility in typical adolescents (TA) and those with AIS [1–4]. Of these studies, none investigate mobility differences found in segments of the spine and instead rely on measures of thoracic and lumbar mobility as a whole to characterize mobility differences cause by AIS.

In this study, spinal mobility was measured during six sagittal and coronal plane bending activities in the thoracolumbar spine of adolescents with and without AIS. The purpose of this pilot study was to define the spinal mobility differences caused by AIS. It was hypothesized that the AIS group will exhibit reduced mobility in all modes of bending compared to the TA group.

## Methods

Eleven adolescents with right thoracic AIS and eleven age- and gender-matched TA were included this study. Subjects who met the inclusion criteria were recruited at scoliosis clinic visits. Inclusion criteria included diagnosis of adolescent idiopathic scoliosis at age ten or later, primary Cobb angle of at least 15°, and no previous history of spinal surgery,. Subjects were excluded if they had any musculoskeletal diagnoses other than AIS. For this study, only scoliosis patients with Lenke type 1 curves (right thoracic curves) were included to mitigate the effect of curve location and type on the outcomes of the study. Eight subjects in each group were female. The average Cobb angle of the primary curve was 48° ± 12° for the scoliosis group. This study was approved by the Institutional Research Board at the University of Kansas-Lawrence and University of Kansas-Medical Center and written and informed consent and assent was obtained for all subjects.

A Trakstar system (Ascension Technologies, Burlington, VT) was used to collect position data at the manubrium, T1, T3, T6, T10, L1, L3, and S1. Participants were instructed the goal was to move to their maximal bent position at a self-selected speed for each of six full motion tasks. The tasks were flexion (F), extension (E), left lateral bending (LLB), right lateral bending (RLB), left 45° anterior-lateral flexion (L45), and right 45° anterior-lateral flexion (R45), as depicted previously [5]. Additional instructions were given to reduce out of plane motion. Tasks were demonstrated and participants were allowed to practice before data collection. Of the five trials for each task, the last trial was used for analysis to allow for the viscoelastic effect to stabilize during testing. Scoliosis curve information for each subject was collected at the time of motion data collection by an orthopedic surgeon (DB) with over 20 years of experience and managed using REDCap electronic data capture tools hosted at the University of Kansas-Lawrence [6].

With the position data, seven angles were calculated as previously described: upper thoracic angle (UT) from T1-T3, mid thoracic angle (MT) from T3-T6, lower thoracic angle (LT) from T6-T10, thoracolumbar angle (TL) from T10-L1, upper lumbar angle (UL) from L1-L3, and thoracic curvature angle from T1-L1 [5]. The normalized range of motion (nROM) was calculated by subtracting the standing position from the maximally bent position and dividing by the number of functional spinal units for each segmental region. All calculations were performed using customized MATLAB (MathWorks, Natick, MA) programs.

Statistical tests were used to determine the mobility differences between the AIS and TA group. A Mann–Whitney test was used to determine statistical differences in the ROM of the AIS and TA groups. Statistical analysis was not conducted when either group had less than five subjects. Although the use of statistical corrections remains controversial, none of the analyses are truly independent; therefore, a statistical correction was not used [7, 8]. All statistical procedures were performed with a significance level of α = 0.05.

## Results

The AIS group had an average age of 15.1 ± 2.0 years, an average height of 1.58 ± 0.18 meters (m), and an average weight of 56.1 ± 15.1 kilograms (kg) while the TA group had an average age of 15.2 ± 2.2 years, an average height of 1.62 ± 0.12 m, and an average weight of 55.2 ± 10.8 kg. The control and scoliosis groups had statistically similar ages, heights, and weights (*p* > 0.05). Both groups demonstrated statistically symmetric mobility in symmetric motion tasks (*p* > 0.05). It was hypothesized that the TA group would experience greater mobility than the AIS group. The results from this study did not support the hypothesis. Instead it seems the AIS group frequently demonstrates greater mobility than the TA group in the thoracolumbar region. While data was collected for all 11 subjects from each group, some mobility results could not be calculated as sensors occasionally exceeded the collection volume in flexion and flexion-type tasks. The following statistical results were obtained from trials where all data was recorded within the collection volume.

**Fig. 1:**
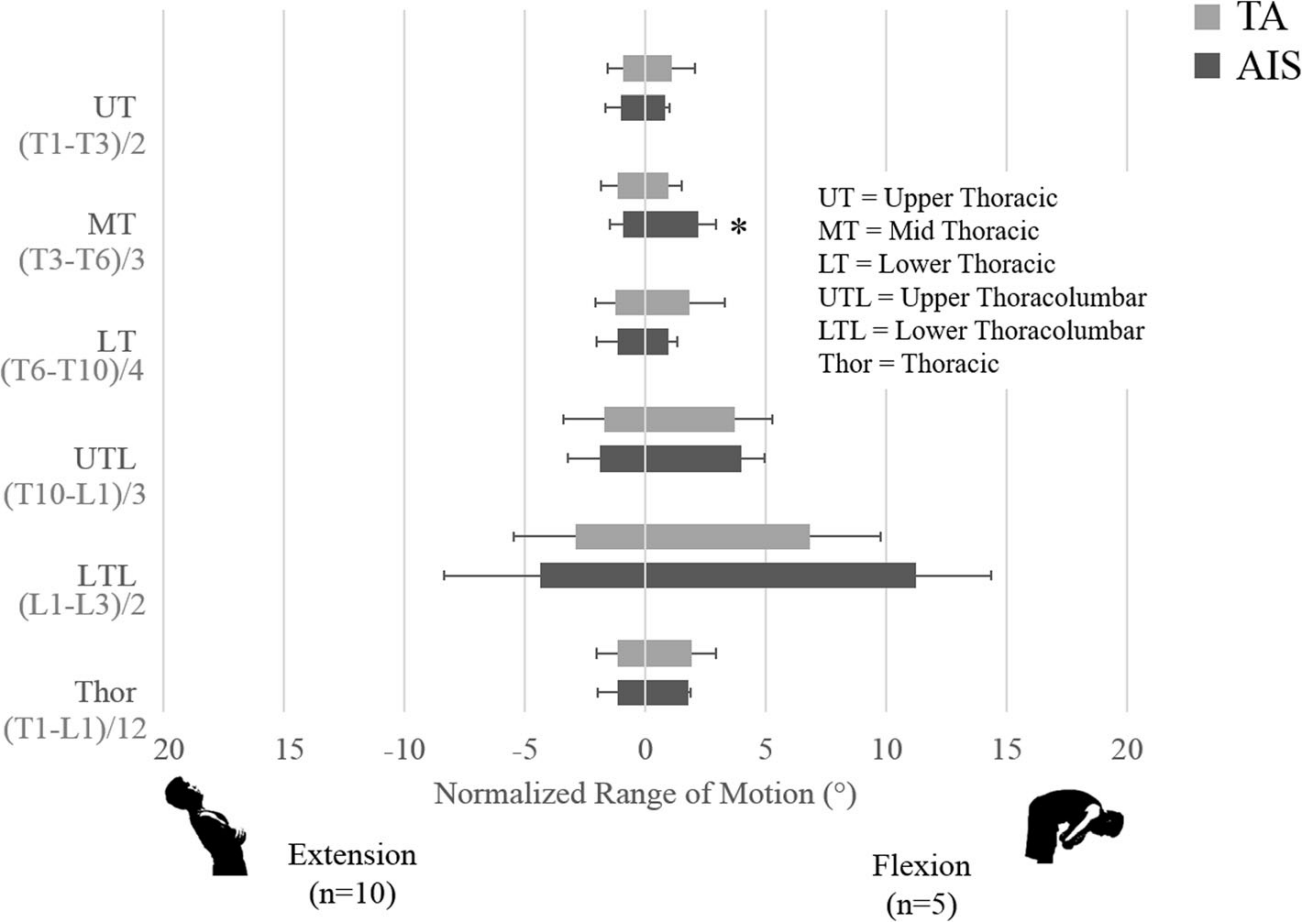
Comparison of functional spine unit normalized ROM of thoracic and thoracolumbar segments of the AIS group to the same segments in the TA group during sagittal plane tasks (flexion and extension). The asterisk denotes the significantly greater nROM in the AIS group compared to the TA group in mid thoracic and upper lumbar motion (*p* = 0.01 *p* = 0.07) during flexion

**Fig. 2:**
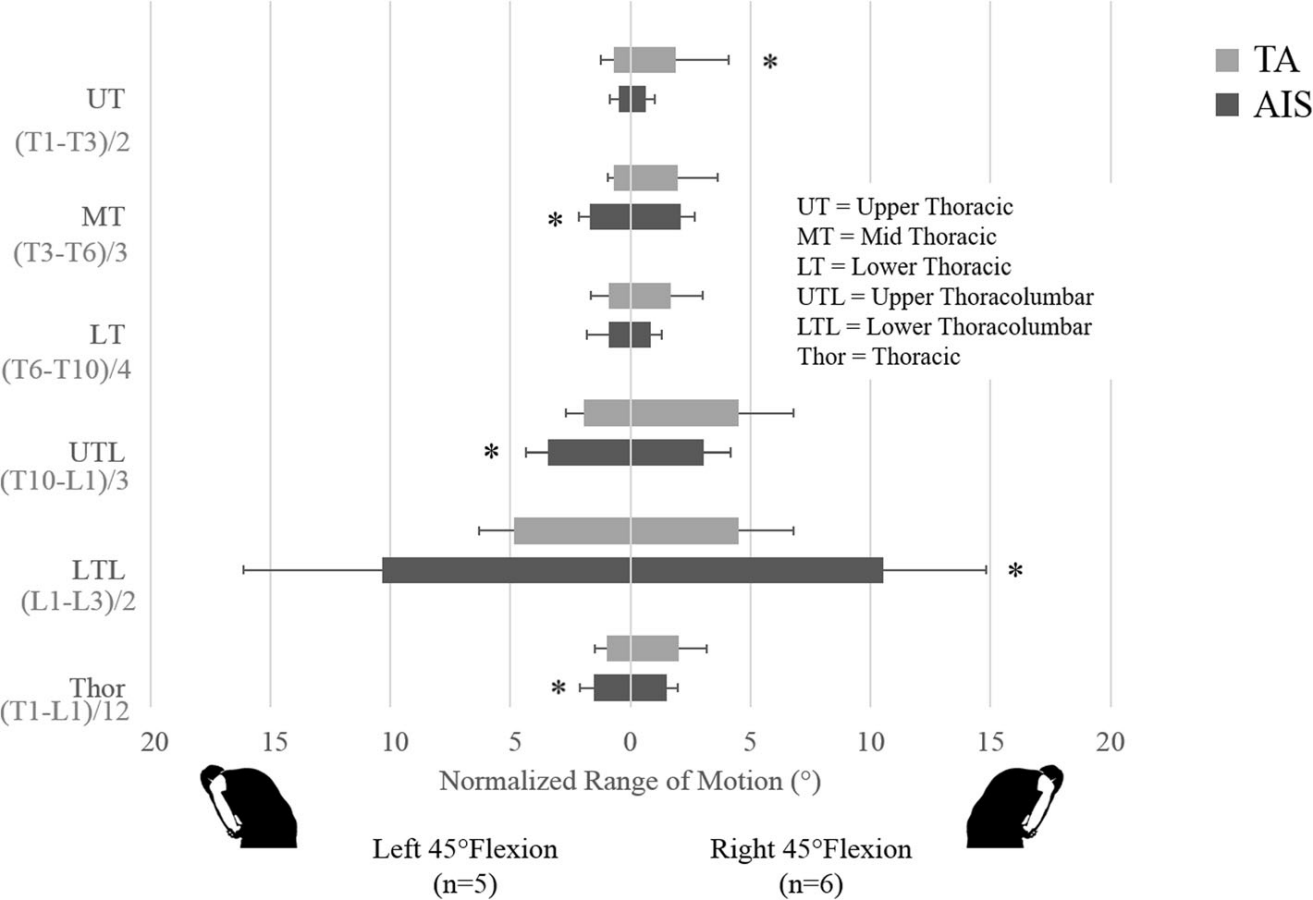
Comparison of functional spine unit normalized ROM of thoracic and thoracolumbar segments of the AIS group to the same segments in the TA group during left and right 45° anterior-lateral flexion. The asterisk denotes the significantly greater nROM in the AIS group in mid thoracic (*p* = 0.01), thoracolumbar (*p* = 0.02), and thoracic (*p* = 0.04) motion during L45 and significantly greater nROM in the TA group in upper thoracic motion during R45 (*p* = 0.02)

While no significant differences were found during extension, consistent mobility patterns can be seen during the flexion and flexion-type tasks. During flexion (Fig. 1), the AIS group was more mobile in the mid thoracic region and trended towards increased mobility in the upper lumbar segmental region (*p* = 0.01, *p* = 0.07). In L45 (Fig. 2), the AIS group had greater mobility than the TA group in the mid thoracic, thoracolumbar, and thoracic segmental regions (*p* = 0.01, *p* = 0.02, *p* = 0.04). In R45 (Fig. 2), the AIS group had greater mobility than the TA group in upper lumbar segmental region (*p* = 0.02). One interesting result of note is that the TA group had greater mobility than the AIS group in the upper thoracic segmental region during R45 (*p* = 0.02). Power for sagittal plane analyses ranged from 2.6 to 36.2 for non-significant comparisons and from 53.6 to 98.7 for significant comparisons.

**Fig. 3:**
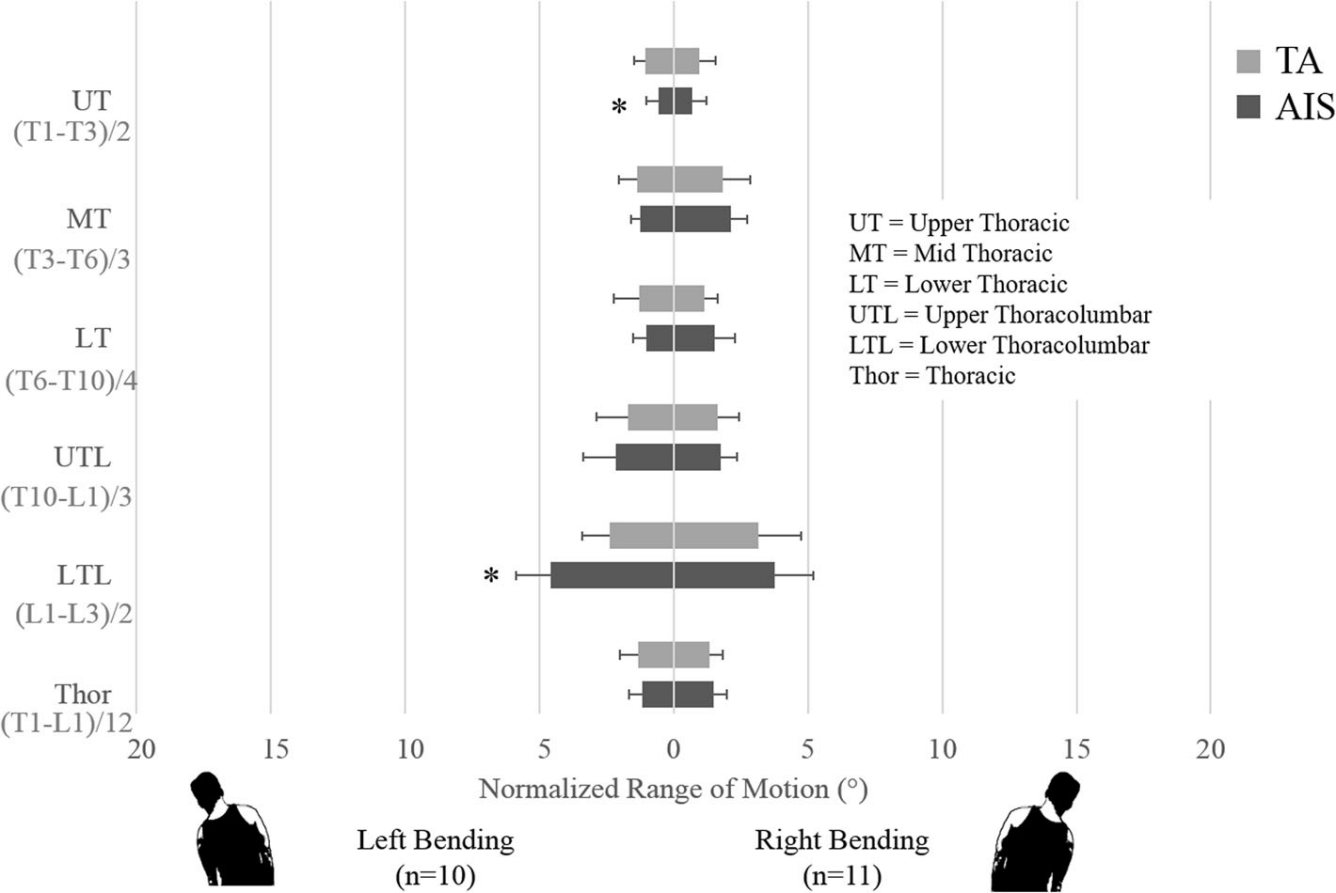
Comparison of functional spine unit normalized ROM of thoracic and thoracolumbar segments of the AIS group to the same segments in the TA group during coronal plane tasks (left and right lateral bending). The asterisk denotes the significantly greater nROM in the AIS group compared to the TA group in upper lumbar motion (*p* < 0.01) and significantly lower nROM in upper thoracic motion (*p* = 0.02) during left lateral bending

Mobility in the lateral bending tasks follows slightly different patterns (Fig. 3). The only instance where the TA group had greater mobility than the AIS group was in the upper thoracic region during left lateral bending (*p* = 0.02). However, in the same bending task, the upper lumbar mobility was greater for the AIS group (*p* < 0.01). In RLB, there were no significant mobility differences between the two groups. As all of the AIS participants have right thoracic curves, the asymmetric mobility results are to be expected. Power for coronal plane analyses ranged from 5.1 to 30.4 for non-significant comparisons. Power of significant comparisons was 72.2 for UT and 98.9 for UL during LLB.

## Discussion

The goal of this research was to characterize the spinal mobility differences caused by AIS, as this has not been done before with segmental detail. It was hypothesized that the AIS group would exhibit reduced mobility in all modes of bending compared to the TA group, but this was not supported. In almost all cases, the mobility of the AIS group was statistically equivalent or significantly greater than the mobility of the TA group.

Mobility values of the current study are compared to literature values for scoliotic adolescents and typical adolescents in Table 1. The mobility values for extension were similar across studies but those presented for flexion and lateral bending were smaller than those presented in the other studies for both TA and AIS populations. Mellin and Poussa [9] use an inclinometer method that has been shown to have a 15° range of motion difference and no correlation with values calculated by the electrogoniometer method, as was used in this study. While these studies shown in Table 1 represent the best comparisons available within the current literature, these studies did not collect data, constrain motion, or select participants with the same methodologies as the current study. Because of the differences in methodologies, differences in mobility outcomes were to be expected.

**Table 1:** Thoracic mobility in control and scoliosis subjects

Group	Author	Flexion	Extension	Left Bending	Right Bending
Control	Galvis et al.	23.2 (11.8)	14.1 (10.1)	15.9 (8.2)	21.9 (12.2)
	Poussa et al.	62.0 (9.1)	−3.3 (14.1)	34.1 (7.3)	32.2 (7.0)
	Mellin and Poussa	62.2–70.3	−4.0–13.0	65.8–82.6	
	Mellin et al.	62.0–69.2	−3.3–-2.6	34.1–37.2	32.2–35.5
Scoliosis	Galvis et al.	18.1 (6.0)	11.4 (9.4)	13.1 (5.4)	16.8 (5.4)
	Poussa et al. (G1)	58.6 (8.7)	18.2 (14.5)	35.2 (7.9)	33.6 (9.8)
	Poussa et al. (G2)	59.9 (11.1)	15.9 (15.3)	34.2 (7.5)	32.6 (8.4)
	Poussa et al. (G3)	50.9 (12.6)	16.5 (19.1)	37.5 (7.8)	25.4 (12.5
	Rahmatalla et al.	28.8	12.1	30.2	28.4

Values presented are mean range of motion values with standard deviation values presented in parentheses where available. The number of subjects varied by bending mode in Galvis et al.: Flexion (*n* = 5), extension (*n* = 10), and left and right lateral bending (*n* = 11). The groups presented for Poussa et al. represented divisions by Cobb angle, with Group 1 having <25° Cobb angles, Group 2 having 25–35° Cobb angle, and Group 3 having Cobb angles >35°

Near the primary curve apex in the AIS group, it was expected the spine would be more rigid than a typical spine. While there was no significant difference between the mobility of the groups, the average range of motion was lower in the scoliosis group compared to the control. One study found the four periapical spinal units experience “structural tethering” where the spinal units demonstrate decreased range of motion [10]. This same effect could be causing the non-significantly lower mobility in the AIS group since the apical effects could have been dampened by the inclusion of “non-tethered” individual motion levels in the lower thoracic region. While structural tethering at the apex was not definitively demonstrated in this study, it has been shown in similar studies and could be the underlying cause of the mobility assessed here but further research is needed.

Results indicate the AIS group had increased nROM in the periapical regions of the spine, particularly in flexion and flexion-type tasks. Many possibilities exist to explain this phenomenon. The segments could be more mobile due to a compensation for reduced mobility near the apex, hypokyphosis in the thoracic spine (though degree of kyphosis was unknown) which would allow for improved rotation about the spinal column, or hyperlaxity of the spine in scoliosis patients which could contribute to deformity progression. The AIS group had greater mobility in the MT region during flexion, in MT, TL, and thoracic during left anterior-lateral flexion, and in UL during right anterior-lateral flexion. The AIS group had significantly higher UL in left lateral bending. In some bending modes, significant mobility differences were seen between the lower thoracic (apical) region and the periapical regions (*p* < 0.05). Without controlling for thoracic kyphosis and with some studies indicating those with scoliosis are no more flexible than their non-pathologic counterparts, compensatory motion may be the mechanism causing the increased mobility. While these results indicate greater mobility above and below the theoretically tethered apical region, this contradicts related findings which indicate mobility above and below long fusions is significantly reduced [11–13]. Since previous research does not agree with the current findings, further study is needed to confirm the increased mobility and its cause.

Other research has shown that thoracic mobility in an AIS population is rarely different compared to controls [1–4]. In this pilot study, there was no statistical difference between the two groups for overall thoracic flexion but there were significant differences in smaller segments of the spine. This showed evaluating thoracic and thoracolumbar mobility to the segmental level is necessary to fully characterize thoracic mobility in an AIS population, as full segmental analyses alone can miss significant mobility differences.

Although there were innovative aspects of this work, there were several limitations to consider as well. Existing three dimensional images were not available; therefore, a three dimensional description of the deformity, including degree of kyphosis and axial rotation, was unknown. Though the current study was designed to be the first to investigate axial rotation in the thoracic spine in an AIS population, the Euler method produced significant data variations when applied to axial rotation. Therefore, spinal motion was not compared in the three primary modes of bending, which would have yielded a three dimensional characterization of adolescent spinal motion. Despite research indicating brace wear can affect curve flexibility, the group was not sub-divided to accommodate for this effect, which is a limitation of the study [14]. This study design did not control for age, Risser grade, or curve severity, which have the potential to effect mobility outcomes in this population and therefore is a limitation. Motion effect from effort, diurnal, sensor placement, soft tissue, and selection variability can obfuscate the true motion and true differences between groups. Trials were the sensors exceeded the collection volume were excluded, which may have eliminated some trials from taller and more flexible participants. With such a small sample size and low power, it was difficult to discern significant differences between groups. Future research should be designed to mitigate against these limitations.

This pilot study was designed to characterize the spinal mobility differences caused by AIS. As gender was not found to have a significant effect on curve flexibility, a mixed gender population was used [15]. Although no gender differences were noted in curve flexibility, subjects were age and gender matched across groups to eliminate any possible cofounding effects. Because curve location and direction affects flexibility, only right thoracic curves with apices between T6-T10 were chosen [2, 16–19]. The age range was limited to isolate the pubertal phase in adolescent subjects to investigate motion differences prior to skeletal maturity. Of the five trials collected for each task, the last trial was used to allow for the viscoelastic effect to stabilize during testing and fatigue effect was not expected as the trials had a high level of repeatability (*r* > 0.9) for all measures in all tasks.

Very little research has focused on comparing spinal function, as measured by spinal mobility, in adolescents both with and without AIS. Of these studies, only one presented range of thoracic mobility and none provided information about near apex mobility [2]. While scoliosis affects large portions of the spine, the deformity varies throughout its length and greatly affects thoracic and lumbar biomechanics. This pilot study was the first to examine spinal function in segmental regions of the thoracic and thoracolumbar spine in adolescents with and without AIS. This investigation shed light on mobility differences caused by the deformity and opened the door for further exploration in this area.

Future work could expand on the research in this study. Three dimensional characterization of the posture and motion would be beneficial. Future studies should control for skeletal maturity and curve severity. As shown by the low power in this study, a larger number of participants would be needed to discern significant mobility differences. This would allow for investigation into segmental mobility differences between scoliosis and control groups with the ability to discern significant differences and isolate the causes of these mobility differences.

## Conclusion

Participants with AIS did not have reduced range of motion in sagittal or coronal motion. On the contrary, the AIS group often had a greater range of motion, especially in segments directly above and below the apex. This indicates the scoliotic spine is flexible and may compensate for any “structural tethering” seen near the apex of curvature. Further work should be pursued to explore the causes of the mobility effect near the apex.
